# Alterations in Choline Metabolism in Non-Obese Individuals with Insulin Resistance and Type 2 Diabetes Mellitus

**DOI:** 10.3390/metabo14080457

**Published:** 2024-08-18

**Authors:** Haya Al-Sulaiti, Najeha Anwardeen, Sara S. Bashraheel, Khaled Naja, Mohamed A. Elrayess

**Affiliations:** 1 Department of Biomedical Sciences, College of Health Sciences, QU Health, Qatar University, Doha P.O. Box 2713, Qatar; haya.alsulaiti@qu.edu.qa; 2 Biomedical Research Center, Qatar University, Doha P.O. Box 2713, Qatar; n.anwardeen@qu.edu.qa (N.A.); khaled.naja@qu.edu.qa (K.N.); 3 College of Medicine, QU Health, Qatar University, Doha P.O. Box 2713, Qatar; sara.bashraheel@qu.edu.qa

**Keywords:** phospholipid metabolites, choline phosphate, glycerophosphoethanolamine, choline, glycerophosphorylcholine (GPC), trimethylamine N-oxide choline, metabolomics, non-obese, insulin resistance, type 2 diabetes mellitus

## Abstract

The prevalence of non-obese individuals with insulin resistance (IR) and type 2 diabetes (T2D) is increasing worldwide. This study investigates the metabolic signature of phospholipid-associated metabolites in non-obese individuals with IR and T2D, aiming to identify potential biomarkers for these metabolic disorders. The study cohort included non-obese individuals from the Qatar Biobank categorized into three groups: insulin sensitive, insulin resistant, and patients with T2D. Each group comprised 236 participants, totaling 708 individuals. Metabolomic profiling was conducted using high-resolution mass spectrometry, and statistical analyses were performed to identify metabolites associated with the progression from IS to IR and T2D. The study observed significant alterations in specific phospholipid metabolites across the IS, IR, and T2D groups. Choline phosphate, glycerophosphoethanolamine, choline, glycerophosphorylcholine (GPC), and trimethylamine N-oxide showed significant changes correlated with disease progression. A distinct metabolic signature in non-obese individuals with IR and T2D was characterized by shifts in choline metabolism, including decreased levels of choline and trimethylamine N-oxide and increased levels of phosphatidylcholines, phosphatidylethanolamines, and their degradation products. These findings suggest that alterations in choline metabolism may play a critical role in the development of glucose intolerance and insulin resistance. Targeting choline metabolism could offer potential therapeutic strategies for treating T2D. Further research is needed to validate these biomarkers and understand their functional significance in the pathogenesis of IR and T2D in non-obese populations.

## 1. Introduction

Phospholipids are crucial cellular membrane lipids which form lipid bilayers. These membrane lipids form barriers between the cell and the environment and between different cellular compartments and are precursors of multiple signaling molecules. Alterations in their homeostasis are associated with the pathogenesis of numerous diseases. Choline, glycerophosphoethanolamine, and glycerophosphorylcholine (GPC) are important constituents of phospholipids [1,2,3,4].

The association of choline, glycerophosphoethanolamine, and GPC with insulin resistance has been investigated in several studies. High dietary choline and betaine intake has been associated with lower levels of insulin resistance [5]. Another study investigating the association between dietary choline and betaine intake and the risk of type 2 diabetes risk indicated sex-based differences in associations. Among male participants, dietary choline or betaine intake was not associated with the risk of type 2 diabetes, whereas among female participants, a trend toward a modestly higher risk of type 2 diabetes was observed in the highest quartile compared with the lowest quartile of dietary choline intake [6]. Another study has reported that choline supplementation induces glucose and insulin intolerance in mice through modulating plasma glucagon and its action in the liver. These findings suggest a potential link between choline metabolism and hepatic insulin resistance [7]. Choline metabolism plays an important role in the development of IS, IR, and T2D. A study found that higher choline intake was associated with lower T2D risk among men in eastern Finland. However, the relationship between choline and T2D risk may differ by sex [8].

Advancements in metabolomics techniques, particularly in mass spectrometry (MS) technologies, have enabled new discovery of new metabolic factors that influence the progression of diseases, including those in metabolically healthy and pathologically obese groups with insulin resistance and T2D [9,10]. Recent evidence has indicated a metabolomics pattern for T2D by identifying metabolites significantly unchanged between lean and obese groups and significantly dysregulated in the T2D group compared with both lean and obese groups. The findings have revealed metabolites associated with insulin resistance and obesity, including phospholipids, thus providing insights into the metabolic changes linked to disease development and progression [11]. 

We have previously identified phospholipid metabolites, including choline, glycerophosphoethanolamine, and GPC, as potential novel biomarkers of obesity-associated insulin sensitivity when compared with obese insulin resistant and type 2 diabetes mellitus (T2D) individuals. These findings have suggested potential diagnostic and therapeutic applications of these metabolites [12]. The novelty of this study lies in its unique approach to understanding the relationship between phospholipid metabolites and insulin resistance in a non-obese population. To close the existing knowledge gap, the aim of this study was to perform an untargeted metabolomics analysis of blood samples in a larger cohort from Qatar Biobank (QBB) in different body mass index (BMI) groups to investigate the association of phospholipid metabolites with IR in non-obese participants. 

## 2. Materials and Methods

### 2.1. Data Source and Study Participants

This study used data from QBB, which compiles comprehensive data on Qatari citizens and long-term residents (≥15 years) who are 18 years of age or older. The QBB’s database contains a wide range of information, including basic personal details and extensive health data such as BMI, blood pressure, blood test results, diabetes history, medication details, and metabolomic profiles, including analysis of more than 1000 metabolites. The data collection and analyses were conducted at the central laboratory of Hamad Medical Corporation, which is certified by the College of American Pathologists. A BMI > 30 is an exclusion factor for this study. A threshold of 1.85 was used as the cut-off point (75th percentile) to dichotomize participants into non-obese insulin-sensitive (IS) and insulin-resistant (IR) groups [13]. Individuals with T2D were categorized based on HbA1C level > 6.5% or glucose ≥7 mmol/L and according to the physician’s diagnosis based on established clinical guidelines, which included a thorough review of the patients’ histories and medical records.

### 2.2. Metabolomics

Metabolomic profiling was performed using Metabolon’s platform, according to standardized protocols. A Waters ACQUITY ultra-performance liquid chromatography (UPLC) instrument (Waters Corporation, Milford, MA, USA) coupled to a Q-Exactive high-resolution/accurate mass spectrometer (Thermo Fisher Scientific, Waltham, MA, USA) was used. This setup included a heated electrospray ionization (HESI-II) source and an Orbitrap mass analyzer, operating at a mass resolution of 35,000. Detailed information regarding the liquid chromatography–mass spectrometry (LC–MS) techniques used herein has been described in prior publications [14]. In brief, the serum samples were initially processed through methanol extraction to remove proteins. The extracted samples were then divided into five parts: two parts were analyzed with different reverse-phase UPLC-MS/MS techniques with positive ion mode electrospray ionization (ESI), one part was analyzed with reverse-phase UPLC-MS/MS with negative ion mode ESI, another part was analyzed with hydrophilic interaction chromatography (HILIC)-UPLC-MS/MS with negative ion mode ESI, and the final part was reserved as a backup sample. The raw data were processed, and peak identification and quality control were performed with the specialized equipment and software provided by Metabolon [15]. The identification of compounds was achieved by comparison with a reference library comprising more than 3300 known standards or consistently present unknown substances. This extensive library comprised a collection of commercially available purified standard compounds. For each examined compound, matches from the library were carefully reviewed for every sample and modified as necessary [16].

### 2.3. Statistical Analysis

The subjects were matched optimally based on their propensity scores using a propensity scores model containing age and sex to ensure balance between IS, IR, and T2D participants, and matched data were used for subsequent analysis. Clinical data were checked for normality using the Shapiro–Wilk test followed by the Kruskal–Wallis test to compare the three groups (IS, IR, and T2D) and Student’s *t*-test/Mann–Whitney U test to compare the mean/median between the IS and IR+T2D groups. Metabolomics data were subjected to inverse rank normalization prior to statistical analysis. Multivariate principal component analysis (PCA) and orthogonal partial least-discriminant analysis (OPLS-DA) was performed to evaluate the difference in the metabolic profile between the study groups (Supplementary Figure S1). PCA was initially used to visualize the data and assess the overall separation between groups. Following this, OPLS-DA was applied to identify specific metabolites that significantly differed between groups.

Univariate analysis was performed via linear regression taking each metabolite as response variable and groups (IS, IR, and T2D) as the predictor variable. In the first model, the group was continuous to represent the different stages of disease progression [IS (1) → IR (2) → T2D (3)], and the second model included the group as categorical variable to confirm previously published study [12] in a non-obese cohort. The model also contained confounding factors such as age, sex, body mass index (BMI), and principal components 1 and 2 (PC1 and PC2) from the PCA. Spearman’s correlation was conducted to investigate the association between the significant metabolites and clinical traits. All statistical tests were performed using R software (version 4.2.1) and SIMCA (version 18.0.0).

## 3. Results

### 3.1. General Characteristics of Study Participants in the Non-Obese Cohort

The non-obese cohort included IS (n = 236), IR (n = 236), and T2D (n = 236) individuals aged <50 years. Due to propensity score matching, the sex distribution was similar between the IS and IR+T2D groups with approximately 62% males and 38% females in the IS group and 65% males and 35% females in the IR+T2D group. The average age of participants in both groups was also similar with a median of 45 years in the IS group and 46 years in the IR+T2D group. The average systolic blood pressure and the average pulse rate were higher in the IR+T2D than the IS group, with a *p*-value of 0.039 and 0.000, respectively. HOMA-IR (a measure of insulin resistance), insulin levels, glucose levels, HBA1c (a marker of long-term blood glucose control), C-Peptide (a marker of insulin production), triglyceride levels, and waist-to-hip ratio (a marker of central obesity) were higher in the IR+T2D group than the IS group, with a significant *p*-value of ≤0.001. Free thyroxine and HDL cholesterol levels were slightly higher in the IS group than the IR+T2D group, with a *p*-value of 0.019. Alkaline phosphatase levels, AST (GOT), and total bilirubin levels did not significantly differ between groups (Table 1).

### 3.2. Phospholipid Metabolites Associated with Different Stages of Disease

**Figure 1:**
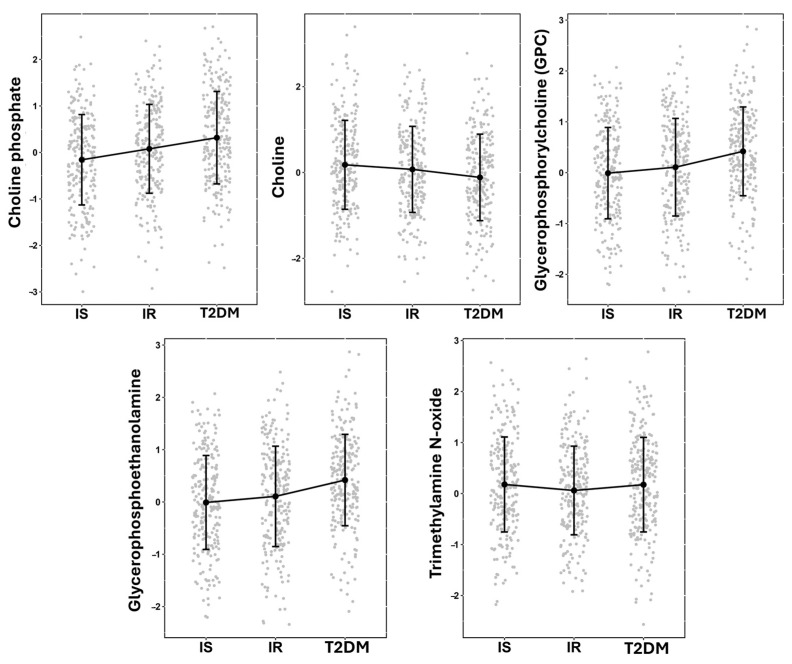
Jitter plot of significant metabolites, showing the means and standard deviations. The figure also features a line plot for visualizing trends among the means of the IS, IR, and T2D groups. *Y*-axis represents the normalized value of metabolites.

A linear model was used to assess the significance of phospholipid metabolites associated with different stages of disease in non-obese individuals by comparing IS vs. IR vs. T2D. Five metabolites exhibited significant differences associated with disease progression (Table 2): choline phosphate, glycerophosphoethanolamine, choline, glycerophosphorylcholine (GPC), and trimethylamine N-oxide. Figure 1 demonstrates patterns of increased choline phosphate, GPC, and glycerophosphoethanolamine or decreased of choline metabolites in the studied groups.

### 3.3. Phospholipids Metabolites Differentiating IS from IR+T2D

Non-targeted metabolomics analysis of serum samples from the 708 participants was performed to identify metabolites significantly differentiating individuals with IS vs. IR+T2D to identify a metabolic signature of non-obesity-associated insulin resistance and T2D. The analysis identified metabolites associated with phospholipid metabolism, specifically choline phosphate, choline, trimethylamine N-oxide, and glycerophosphoethanolamine.

Linear regression analysis (Table 3) estimated a positive effect size of 0.299 and 0.205 for choline phosphate and glycerophosphoethanolamine, respectively. In contrast, choline and trimethylamine N-oxide exhibited negative effect sizes of −0.303 and −0.215.

**Figure 2:**
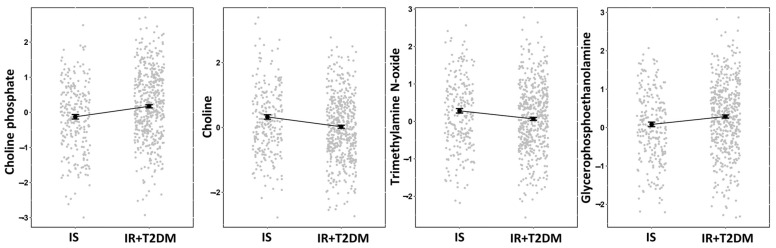
Jitter plot of significant deferential metabolites depicting the adjusted means derived from a linear regression model. The means were calculated with emmeans package in R and includes corresponding standard errors. The figure also features a line plot for visualizing trends in the means between the IS and IR+T2D groups. *Y*-axis represents the normalized value of metabolites.

To visualize the trends among the means of these significant metabolites, we constructed a jitter plot (Figure 2) displaying the adjusted means derived from a linear regression model, along with their corresponding standard errors (SEs). Additionally, a line plot is included in Figure 2 to aid in visualization of the trends between the IS and IR+T2D groups. Figure 2 illustrates the differences in metabolite levels between the IS and IR+T2D groups. Choline phosphate and glycerophosphoethanolamine had higher levels in the IS group than the IR+T2D group. In contrast, choline and trimethylamine N-oxide had lower levels in the IS group than the IR+T2D group.

### 3.4. Other Significant Phospholipid-Associated Metabolites Significantly Differentiating IS vs. IR+T2D in Non-Obese Participants

We performed linear regression analysis to identify the presence of significant phospholipid-associated metabolites in non-obese participants. Table 4 provides a comprehensive overview of the findings. The analysis revealed the presence of various types of phospholipids, including phosphatidylcholine, phosphatidylethanolamine, and phosphatidylinositol. 

In the phosphatidylcholine sub-pathway, several metabolites significantly differed between groups, such as 1-palmitoyl-2-palmitoleoyl-GPC (16:0/16:1), 1-palmitoyl-2-dihomo-linolenoyl-GPC (16:0/20:3n3 or 6), and 1-myristoyl-2-arachidonoyl-GPC (14:0/20:4). In the phosphatidylethanolamine sub-pathway, significant metabolites included 1-palmitoyl-2-arachidonoyl-GPE (16:0/20:4), 1-stearoyl-2-linoleoyl-GPE (18:0/18:2), and 1-palmitoyl-2-docosahexaenoyl-GPE (16:0/22:6). Finally, in the phosphatidylinositol sub-pathway, significant metabolites included 1-palmitoyl-2-arachidonoyl-GPI (16:0/20:4) and 1-palmitoyl-2-oleoyl-GPI (16:0/18:1). These findings contribute to a better understanding of the phospholipid-associated metabolites in non-obese individuals.

### 3.5. Correlation of Significant Metabolites with Mediators of Metabolic Disease

**Figure 3:**
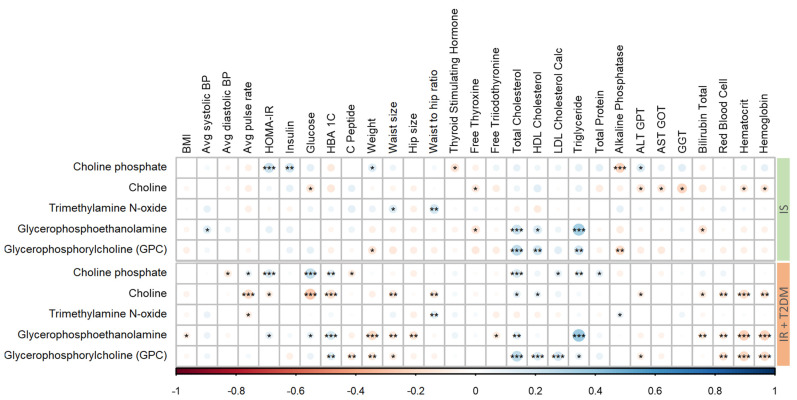
Correlation matrix of significant metabolites with clinical parameters in participants with IS and IR+T2D (***/**/* indicates *p*-values < 0.001/<0.01/<0.05, respectively). Red/blue denotes negative/positive Pearson’s correlation coefficient (r), respectively.

To determine which clinical parameters are most strongly linked to the metabolites that exhibit significant differences across disease groups (as shown in Figure 3), a partial correlation analysis was performed. This statistical technique allows for evaluating the relationship between each metabolite and each clinical parameter while controlling for the potential influence of all other parameters. In the IS group, a positive correlation of total cholesterol with the metabolites glycerophosphoethanolamine and GPC was observed. In contrast, in the IR+T2D group, total cholesterol was positively correlated with choline phosphate and GPC. Choline levels were negatively correlated with the average pulse rate, glucose, and HbA1C (glycated hemoglobin). Moreover, glycerophosphoethanolamine and GPC in IR+T2D participants showed significant positive correlations with hematocrit and hemoglobin.

## 4. Discussion

Phospholipid metabolism plays a central role in the pathogenesis of metabolic diseases, including insulin resistance and T2D [17]. Despite the evidence from clinical studies indicating a link between phospholipids and insulin sensitivity, it remains uncertain whether alterations in phospholipids are the cause or result of insulin resistance. The aim of this study was to investigate the association of phospholipid metabolites with insulin resistance and T2D in non-obese participants.

Our results showed a significant decrease in choline levels with progression from insulin sensitivity to insulin resistance and T2D. Similar results were obtained when the IS group was compared with the combined group (IR+T2D). This is in line with our previous study on obese participants [12] with Lemaitre et al., who showed that choline was associated with greater insulin sensitivity [18], and Gao et al. [19], who reported that dietary choline was negatively correlated with insulin resistance. Moreover, Corbin et al. demonstrated that choline deficiency in humans is associated with liver dysfunction [20]. However, Al-Aama et al. [21] reported increased choline levels in T2D patients.

Our results also showed an increased level of glycerophosphorylcholine (GPC) with metabolic dysfunction progression, accompanied by a significant increase in many phosphatidylcholines. This suggested activated choline metabolism is further corroborated by a significant rise in choline phosphate, the first intermediate in the choline metabolic pathway. In fact, in the Kennedy pathway [22], choline is phosphorylated to choline phosphate (PC) by choline kinase, and then PC is converted to cytidine diphosphate choline (CDP-choline). Then CDP-choline and diacylglycerol are used to produce phosphatidylcholines, which in their turn, are broken down to form GPC by the action of phospholipases.

The activated choline metabolism, characterized by an increase in PC and GPC, has become a hallmark of carcinogenesis and tumor progression, as these metabolites were detected in all tested cancer types [23]. Yet, the interplay between choline metabolism and insulin resistance and T2D is less clearly understood. Zeisel et al. [24] demonstrated that mice with deletions in one of several genes involved in choline metabolism exhibited enhanced insulin sensitivity. Moreover, Kumar et al. showed that the upregulation of exosomal PC contributes to insulin resistance in lean mice [25], and He et al. [26] reported that reduced polyunsaturated PC in adipocyte plasma membranes increased insulin sensitivity. PC and GPC play crucial roles in cellular membrane composition and signaling pathways, influencing the overall metabolic health. PC contributes to membrane integrity and fluidity, participates in lipid metabolism, and plays a crucial role in liver function and cellular signaling. GPC acts as an osmolyte, a choline reservoir, and supports neuroprotection and signal transduction. The balance and function of PC and GPC are essential for maintaining cellular health and overall metabolic balance, with disruptions potentially leading to various metabolic disorders [17].

However, in our previous study on participants with obesity [12], metabolic dysfunction progression was associated with decreased levels of GPC. Additionally, Suhre et al. [27] reported lower levels of GPC in patients with T2D when compared to healthy controls. Remarkably, the T2D participants in both studies were obese, suggesting that obesity may be a confounding factor affecting phosphatidylcholines and GPC levels.

Interestingly, our findings also revealed an association between metabolic dysfunction progression and elevated levels of phosphatidylethanolamine, another Kennedy pathway metabolite, as well as its degradation product glycerophosphoethanolamine (GPE). Phosphatidylethanolamine is synthesized de novo via the CDP–ethanolamine pathway analogous to CDP–choline pathway. Phosphatidylethanolamine can also be converted into phosphatidylcholine by N-methyltransferase in a pathway specific to the liver [28]. Phosphatidylethanolamine is a major component and cell membrane and plays a crucial role in its integrity and fluidity [29]. Low membrane fluidity has been associated with impaired insulin signaling [30], and one way that the cell membrane fluidity can be increased is through decreasing phosphatidylethanolamine [29]. Moreover, alterations in PE and GPE levels can lead to the accumulation of diacylglycerol, a known contributor to insulin resistance [17].

Relatedly, a clinical study involving lean and overweight subjects demonstrated that HOMA-IR was positively correlated with the content of phosphatidylethanolamine and phosphatidylcholine in erythrocyte membranes across the entire study population [31].

Our emerging results showed significantly decreased levels of trimethylamine N-oxide (TMAO) with progression from insulin sensitivity to insulin resistance and T2D. TMAO is a gut microbiota metabolite [32], and its association with insulin resistance and diabetes has been a topic of interest in recent studies, but the results have been inconsistent.

In line with our study, the PREDIMED case-cohort study reported that high plasma TMAO concentrations were associated with a decreased risk of incident diabetes [33]. Moreover, Trøseid et al. [34] documented a two-fold increase in TMAO plasma concentrations accompanied by a decrease in glycated hemoglobin after one year of bariatric surgery which is known to reduce CVD risk. However, conflicting studies have been published. While the results by Lemaitre et al. [18] and Roy et al. [35] do not report any association between TMAO and the increased risk of insulin resistance and incident diabetes, Schugar et al. [36] reported a positive correlation between plasma levels of TMAO and insulin resistance. Additionally, Li et al. [37] and Svingen et al. [38] reported an association between higher serum TMAO with a higher risk of T2D. Given the potential variability in microbiome composition and metabolic pathways across the studied cohorts, the microbial metabolite TMAO may exhibit distinct behaviors and have varying impacts on health outcomes in different populations, which could contribute to the observed inconsistencies in research findings. Further research is warranted to uncover the specific mechanisms and contexts in which TMAO influences insulin resistance and overall human health.

**Figure 4:**
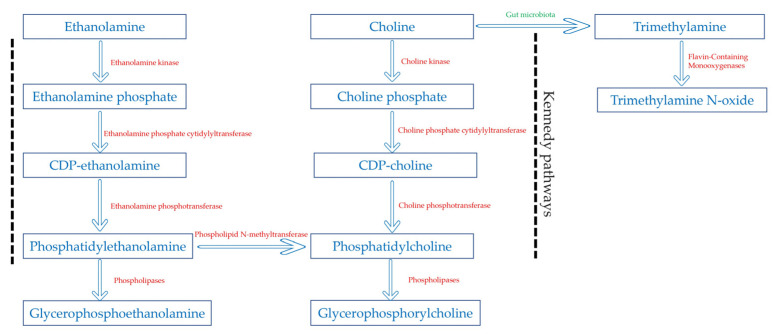
Metabolic pathways involved in this study.

Taken together with existing literature, our results indicate that the progression from insulin sensitivity to insulin resistance and T2D is associated with an increase in choline metabolism and a shift to CDP–choline and CDP–ethanolamine pathways over the TMAO pathway (Figure 4). Our results were further corroborated by the direct correlation of choline phosphate, GPC, and GPE with glucose and HbA1C in the (IS+T2D) group. In contrast, choline exhibited an inverse correlation with these key glycemic parameters. 

Another crucial point to consider is the fatty acid composition of phosphatidylcholines and phosphatidylethanolamines in the cell membrane which may modulate the action of insulin. Our results showed that the metabolic dysfunction progression was associated with phospholipids containing high levels of saturated fatty acids. Indeed, decreased insulin sensitivity correlates with reduced levels of polyunsaturated fatty acids in skeletal muscle phospholipids [17]. Moreover, phospholipids containing saturated fatty acids decrease the aforementioned membrane fluidity and therefore decrease insulin sensitivity [30].

Nevertheless, the interrelationship between insulin sensitivity and phospholipids is a very complex process. It is essential to understand that numerous cellular changes occur in response to insulin stimulation, which can affect phospholipid metabolism. Further investigation is still required in order to obtain more insight into the functional significance of these observations.

## 5. Conclusions

Insulin resistance and T2D in this study were associated in non-obese participants with a shift in choline metabolism characterized by a decrease in choline and trimethylamine N-oxide levels and an increase in phosphatidylcholines and phosphatidylethanolamines and their degradation products. These findings suggest that choline metabolism may be a critical factor in the development of glucose intolerance and insulin resistance and that targeting choline metabolism pharmacologically or through the diet may be a potential therapeutic strategy for the treatment of T2D.

## 6. Limitations

This study has some limitations. First, the data were obtained from QBB, which includes information from Qatari nationals and long-term residents, so the findings may not be generalizable to other populations. Second, the study focused on alterations in choline metabolism in non-obese individuals with insulin resistance and type 2 diabetes mellitus, without investigating other factors such as lifestyle, diet, or genetic predispositions. Finally, the observational nature of this study limits the conclusions of the causal associations between metabolites and diseases. Future longitudinal studies are warranted to further understand the underlying mechanisms that may be driving these observed associations. Additionally, it would be important to conduct studies in diverse populations to ensure the generalizability of these findings.

## Figures and Tables

**Table 1:** General characteristics of participants in the non-obese cohort.

Variables	IS (n = 236)	IR (n = 36)	T2D (n = 236)	*p*-Value (KW)	IR+T2D (n = 472)	*p*-Value (IS vs. IR+T2D)
Sex						
Male	146 (61.86%)	149 (62.3%)	160 (66.9%)		309 (65.47%)	0.390
Female	90 (38.14%)	87 (36.4%)	76 (31.8%)		163 (34.53%)	
Age (years)	45 (39–49.25)	45 (39–49)	49.5 (41–57)	<0.001	46 (40–54)	0.009
BMI (kg/m^2^)	26.91 (25.19–28.36)	27.19 (25.43–28.61)	27.1 (25.56–28.58)	0.467	27.19 (25.48–28.6)	0.228
Average systolic BP (mmHg)	114.5 (105–126)	114 (107–123)	119 (112–130)	<0.001	117 (109–127)	0.039
Average diastolic BP (mmHg)	74 (67–82)	75 (68–83)	75.5 (69–81)	0.935	75 (68–81)	0.716
Average pulse rate (beats/min)	65 (59–70)	67 (62–72)	72 (65–79)	<0.001	68 (63–76)	<0.001
HOMA-IR (mmol/L)	1.31 (1–1.57)	3.13 (2.2–4.86)	4.84 (2.42–11.22)	<0.001	3.59 (2.21–7.19)	<0.001
Insulin (pmol/L)	6 (5–7)	13.1 (10–20.02)	13 (7.45–25.75)	<0.001	13 (9–22.4)	<0.001
Glucose (mmol/L)	4.82 (4.6–5.1)	5.26 (4.9–5.7)	7.5 (6.3–9.93)	<0.001	5.8 (5.1–7.5)	<0.001
HBA1c (%)	5.4 (5.1–5.7)	5.5 (5.2–5.7)	6.7 (6–8)	<0.001	5.8 (5.4–6.7)	<0.001
C-Peptide (nmol/L)	1.66 (1.42–2.11)	3 (2.3–4.12)	2.66 (1.73–4.06)	<0.001	2.84 (2.06–4.08)	<0.001
Weight (kg)	74.5 (67.52–80.88)	74.75 (67.45–82.62)	73.05 (66.9–80.5)	0.371	74 (67.18–81.53)	0.939
Waist size (cm)	86 (80.75–92)	88 (82–94)	90 (86–97)	<0.001	89 (83–95)	<0.001
Hip size (cm)	102 (98–106)	103 (99–106)	100 (97–104)	<0.001	101 (98–105.25)	0.198
Waist-to-hip ratio	0.86 (0.79–0.9)	0.87 (0.8–0.92)	0.92 (0.84–0.96)	<0.001	0.89 (0.82–0.94)	<0.001
Free thyroxine (pmol/L)	13.22 (12.28–14.2)	12.7 (11.88–13.7)	13.12 (12.29–14.16)	0.001	12.9 (12.05–13.83)	0.019
Free triiodothyronine (pmol/L)	4.4 (4–4.7)	4.5 (4.1–4.9)	4.4 (4–4.8)	0.076	4.45 (4.1–4.8)	0.137
Total cholesterol (mmol/L)	5.1 (4.4–5.88)	5.26 (4.6–5.85)	4.94 (4.2–5.63)	0.003	5.1 (4.4–5.75)	0.499
HDL cholesterol (mmol/L)	1.35 (1.14–1.61)	1.22 (1.04–1.45)	1.13 (0.94–1.38)	<0.001	1.19 (0.99–1.43)	<0.001
LDL cholesterol (mmol/L)	3.08 (2.52–3.92)	3.18 (2.75–3.94)	3 (2.1–3.6)	0.001	3 (2.37–3.76)	0.259
Triglyceride (mmol/L)	1.1 (0.81–1.48)	1.37 (0.99–2)	1.69 (1.12–2.36)	<0.001	1.5 (1.05–2.2)	<0.001
Total protein	73 (71–76)	73 (70–76)	73 (70–75)	0.146	73 (70–75)	0.182
Alkaline phosphatase (U/L)	65 (54–80)	67 (56–79)	68 (56–81)	0.434	67 (56–80)	0.319
ALT (GPT) (U/L)	19 (14–27.25)	22 (16–33)	21 (16–31)	<0.001	21 (16–32)	<0.001
AST (GOT) (U/L)	18 (15–22)	18 (16–24)	17 (14–22)	0.002	18 (15–23)	0.932
GGT (U/L)	16.5 (13–26)	24 (14–34.5)	23 (15.5–35.5)	0.026	23 (14.25–35)	0.008
GGT_2 (U/L)	23 (17–31)	27 (18.5–37)	28 (18.5–42)	0.032	27 (18.25–38)	0.011
Bilirubin Total (μmol/L)	6.95 (5–9)	6 (4.7–8.75)	6.45 (4.62–9)	0.346	6.2 (4.7–9)	0.229

*p*-value significance level of 0.05 was used. The data are presented as median (IQR) after the Shapiro–Wilk normality test has indicated that it does not follow a normal distribution, and the medians were compared using Mann–Whitney U test and Kruskal–Wallis (KW) test. Percentage/count data were compared using Chi square test. [ALT—Alanine transaminase, AST—Aspartate aminotransferase, BMI—Body mass index, GGT—Gamma-glutamyl transferase, HOMA-IR—Homeostatic Model Assessment for Insulin Resistance, HDL—High-density lipoprotein, LDL—Low-density lipoprotein].

**Table 2:** Phospholipid metabolites associated with metabolic dysfunction progression in non-obese individuals.

Metabolite	Sub-Pathway	Estimate	SE	*p*-Value	FDR
Choline phosphate	Phospholipid Metabolism	0.232	0.045	2.52 × 10^−7^	2.69 × 10^−6^
Glycerophosphoethanolamine	Phospholipid Metabolism	0.186	0.041	7.09 × 10^−6^	5.38 × 10^−5^
Choline	Phospholipid Metabolism	−0.199	0.047	3.08 × 10^−5^	2.06 × 10^−4^
Glycerophosphorylcholine (GPC)	Phospholipid Metabolism	0.139	0.045	2.21 × 10^−3^	8.36 × 10^−3^
Trimethylamine N-oxide	Phospholipid Metabolism	−0.108	0.041	9.01 × 10^−3^	2.82 × 10^−2^

FDR significance of 0.05 was used.

**Table 3:** Results of linear regression analysis to identifying significant phospholipid metabolites between IS and IR+T2D.

Metabolites	Sub-Pathway	Estimate	SE	*p*-Value	FDR
Choline phosphate	Phospholipid Metabolism	0.299	0.077	1.19 × 10^−4^	8.18 × 10^−4^
Choline	Phospholipid Metabolism	−0.303	0.082	2.31 × 10^−4^	1.45 × 10^−3^
Trimethylamine N-oxide	Phospholipid Metabolism	−0.215	0.071	2.55 × 10^−3^	1.18 × 10^−2^
Glycerophosphoethanolamine	Phospholipid Metabolism	0.205	0.071	4.11 × 10^−3^	1.77 × 10^−2^

FDR significance of 0.05 was used.

**Table 4:** Other significant phospholipid-associated metabolites in non-obese participants.

Metabolite	Sub-Pathway	Estimate	SE	*p*-Value	FDR
1-Palmitoyl-2-palmitoleoyl-GPC (16:0/16:1) *	Phosphatidylcholine	0.431	0.068	4.51 × 10^−10^	1.78 × 10^−8^
1-Palmitoyl-2-oleoyl-GPC (16:0/18:1)	Phosphatidylcholine	0.342	0.059	1.12 × 10^−8^	2.95 × 10^−7^
1-Palmitoyl-2-dihomo-linolenoyl-GPC (16:0/20:3n3 or 6) *	Phosphatidylcholine	0.367	0.064	1.6 × 10^−8^	4.21 × 10^−7^
1-Palmitoyl-2-arachidonoyl-GPC (16:0/20:4n6)	Phosphatidylcholine	0.333	0.060	4.17 × 10^−8^	9.55 × 10^−7^
1-Myristoyl-2-arachidonoyl-GPC (14:0/20:4) *	Phosphatidylcholine	0.348	0.064	8.93 × 10^−8^	1.85 × 10^−6^
1-Stearoyl-2-oleoyl-GPC (18:0/18:1)	Phosphatidylcholine	0.278	0.061	6.62 × 10^−6^	7.48 × 10^−5^
1-Myristoyl-2-palmitoyl-GPC (14:0/16:0)	Phosphatidylcholine	0.288	0.071	5.17 × 10^−5^	4.06 × 10^−4^
1-Palmitoyl-2-linoleoyl-GPC (16:0/18:2)	Phosphatidylcholine	0.265	0.073	3.04 × 10^−4^	1.81 × 10^−3^
1-Stearoyl-2-arachidonoyl-GPC (18:0/20:4)	Phosphatidylcholine	0.220	0.063	5.30 × 10^−4^	3.01 × 10^−3^
1,2-Dipalmitoyl-GPC (16:0/16:0)	Phosphatidylcholine	0.198	0.059	7.42 × 10^−4^	4.01 × 10^−3^
1-Palmitoyl-2-arachidonoyl-GPE (16:0/20:4) *	Phosphatidylethanolamine	0.438	0.062	5.76 × 10^−12^	4.56 × 10^−10^
1-Palmitoyl-2-oleoyl-GPE (16:0/18:1)	Phosphatidylethanolamine	0.440	0.067	8.78 × 10^−11^	4.50 × 10^−9^
1-Palmitoyl-2-linoleoyl-GPE (16:0/18:2)	Phosphatidylethanolamine	0.421	0.068	9.41 × 10^−10^	3.28 × 10^−8^
1-Stearoyl-2-oleoyl-GPE (18:0/18:1)	Phosphatidylethanolamine	0.370	0.069	1.23 × 10^−7^	2.39 × 10^−6^
1-Stearoyl-2-arachidonoyl-GPE (18:0/20:4)	Phosphatidylethanolamine	0.357	0.068	2.26 × 10^−7^	4.02 × 10^−6^
1-Stearoyl-2-linoleoyl-GPE (18:0/18:2) *	Phosphatidylethanolamine	0.340	0.075	7.54 × 10^−6^	8.31 × 10^−5^
1-Palmitoyl-2-docosahexaenoyl-GPE (16:0/22:6) *	Phosphatidylethanolamine	0.226	0.064	3.96 × 10^−4^	2.31 × 10^−3^
1-Palmitoyl-2-arachidonoyl-GPI (16:0/20:4) *	Phosphatidylinositol	0.319	0.077	3.50 × 10^−5^	2.90 × 10^−4^
1-Palmitoyl-2-oleoyl-GPI (16:0/18:1) *	Phosphatidylinositol	0.300	0.076	8.53 × 10^−5^	6.24 × 10^−4^
1-Palmitoyl-2-linoleoyl-GPI (16:0/18:2)	Phosphatidylinositol	0.302	0.077	9.11 × 10^−5^	6.62 × 10^−4^
1-Stearoyl-2-arachidonoyl-GPI (18:0/20:4)	Phosphatidylinositol	0.280	0.076	2.32 × 10^−4^	1.45 × 10^−3^

FDR significance threshold of 0.05 was used. * Indicates that Metabolon is confident about the identity of the metabolites, however it is not confirmed based on the standard.

## Data Availability

The datasets used and/or analyzed during the current study are available from the corresponding author on reasonable request.

## Supplementary Materials

The following supporting information can be downloaded at: https://www.mdpi.com/article/10.3390/metabo14080457/s1, Figure S1: Multivariate principal component analysis (PCA) and orthogonal partial least-discriminant analysis (OPLS-DA) analysis to evaluate the difference in the metabolic profile between the study groups.
