# Supplementary material for: Alterations in Choline Metabolism in Non-Obese Individuals with Insulin Resistance and Type 2 Diabetes Mellitus
Source: Metabolites. 2024 Aug 18;14(8):457. doi: 10.3390/metabo14080457 (PMC11356528; doi:10.3390/metabo14080457)
Supplement: Supplementary file 1 [file metabolites-14-00457-s001.zip › metabolites-3123270-supplementary.pdf]

Supplementary Materials.

Supplementary Figure

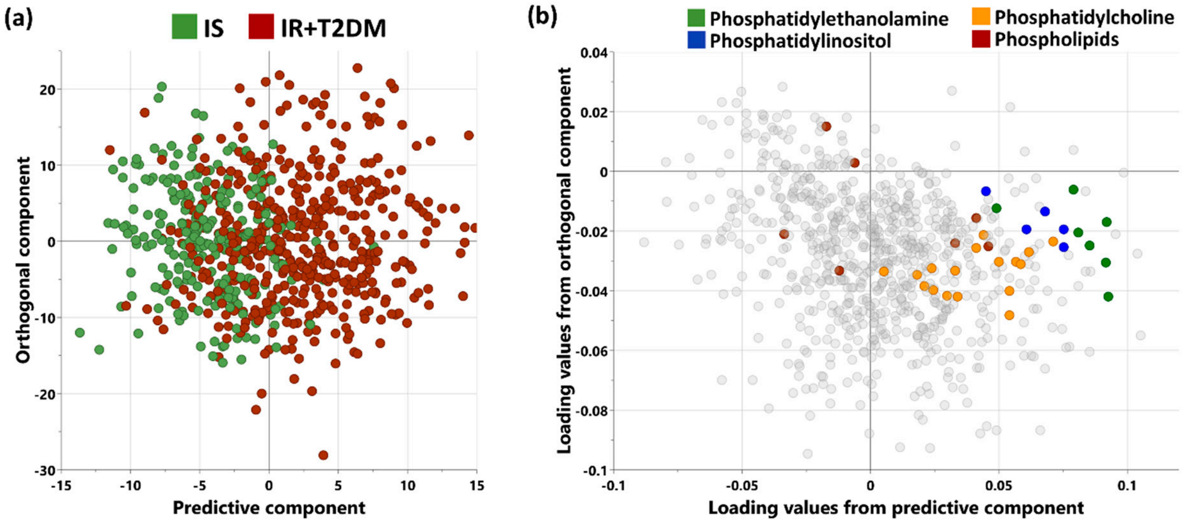

Figure S1. (**a**) Scores and (**b**) loading plot from OPLS-DA. The model parameters are R2Y-35.2% and Q2-30%. CV-ANOVA *p*-value of ≤0.001, indicates that the OPLS-DA model is statistically significant and provides good discriminatory power in distinguishing between the specified groups. Metabolites related to phospholipids are highlighted in the loadings plot.
